# Health Care Students’ Perspectives on Artificial Intelligence: Countrywide Survey in Canada

**DOI:** 10.2196/33390

**Published:** 2022-01-31

**Authors:** Minnie Teng, Rohit Singla, Olivia Yau, Daniel Lamoureux, Aurinjoy Gupta, Zoe Hu, Ricky Hu, Amira Aissiou, Shane Eaton, Camille Hamm, Sophie Hu, Dayton Kelly, Kathleen M MacMillan, Shamir Malik, Vienna Mazzoli, Yu-Wen Teng, Maria Laricheva, Tal Jarus, Thalia S Field

**Affiliations:** 1 Faculty of Medicine University of British Columbia Vancouver, BC Canada; 2 School of Occupational Science and Occupational Therapy Faculty of Medicine University of British Columbia Vancouver, BC Canada; 3 School of Biomedical Engineering University of British Columbia Vancouver, BC Canada; 4 Northern Ontario School of Medicine Thunder Bay, ON Canada; 5 Queen's University Kingston, ON Canada; 6 University of Alberta Edmonton, AB Canada; 7 University of Calgary Calgary, AB Canada; 8 Northern Ontario School of Medicine Sudbury, ON Canada; 9 Faculty of Medicine Dalhousie University Saint John, NB Canada; 10 Temerty Faculty of Medicine University of Toronto Toronto, ON Canada; 11 Vancouver Coastal Health Vancouver, BC Canada; 12 Faculty of Arts University of British Columbia Vancouver, BC Canada; 13 Vancouver Stroke Program Division of Neurology University of British Columbia Vancouver, BC Canada

**Keywords:** medical education, artificial intelligence, allied health education, medical students, health care students, medical curriculum, education

## Abstract

**Background:**

Artificial intelligence (AI) is no longer a futuristic concept; it is increasingly being integrated into health care. As studies on attitudes toward AI have primarily focused on physicians, there is a need to assess the perspectives of students across health care disciplines to inform future curriculum development.

**Objective:**

This study aims to explore and identify gaps in the knowledge that Canadian health care students have regarding AI, capture how health care students in different fields differ in their knowledge and perspectives on AI, and present student-identified ways that AI literacy may be incorporated into the health care curriculum.

**Methods:**

The survey was developed from a narrative literature review of topics in attitudinal surveys on AI. The final survey comprised 15 items, including multiple-choice questions, pick-group-rank questions, 11-point Likert scale items, slider scale questions, and narrative questions. We used snowball and convenience sampling methods by distributing an email with a description and a link to the web-based survey to representatives from 18 Canadian schools.

**Results:**

A total of 2167 students across 10 different health professions from 18 universities across Canada responded to the survey. Overall, 78.77% (1707/2167) predicted that AI technology would affect their careers within the coming decade and 74.5% (1595/2167) reported a positive outlook toward the emerging role of AI in their respective fields. Attitudes toward AI varied by discipline. Students, even those opposed to AI, identified the need to incorporate a basic understanding of AI into their curricula.

**Conclusions:**

We performed a nationwide survey of health care students across 10 different health professions in Canada. The findings would inform student-identified topics within AI and their preferred delivery formats, which would advance education across different health care professions.

## Introduction

### Background

Artificial intelligence (AI) is poised to revolutionize modern health care in the near future. Health care provision, as well as the roles of providers, may be affected by AI through enhanced clinical decision-making, streamlined clinical workflow, improved resource allocation, reduced workloads, and improved efficiency [1-5]. The most prominent current applications of AI in the medical field are in medical imaging analysis [3], particularly with the use of deep learning (DL). DL, a subfield of AI, is defined as “a type of artificial intelligence that uses a layered algorithmic architecture to analyze data” [6]. DL has a wide range of applications and is especially useful for identifying complex yet subtle discriminative patterns in images [3]. Such proficiency is applicable in pattern-centric disciplines of medicine, including radiology, dermatology, and pathology [1,2,7]. As AI continues to evolve, its use is expanding beyond image classification to signal processing in cardiology [8,9] and natural language processing in psychiatry [10] and will continue to grow.

A recent surge in interest in training health care students in AI is reflective of the increasing integration of AI applications in education, research, and clinical care. Among others, the Royal College of Physicians and Surgeons of Canada and the Association of American Medical Colleges have recommended education for health care professionals related to AI, including data provenance and curation, ethics of AI, and critical appraisal and interpretation of AI applications in health care [11-14]. In addition, limited AI exposure has been shown to induce anxiety in undergraduate medical students, affecting their future career decision-making [15,16]. Therefore, exploring the general attitudes and current knowledge base of health care students may be a powerful approach for highlighting areas of need for curriculum decision-makers with respect to AI education [17].

Despite the growing role of AI in health care, the literature on the perspectives of health care students on AI is scant. To date, a few surveys have been conducted on Doctor of Medicine (MD) degree students in Canada [15], the United Kingdom [16], and Germany [18], all of which primarily focused on how students’ perceptions of AI may affect their choice of career in radiology. These studies were limited by their small sample sizes, with sampling performed only at select medical institutions. Furthermore, as the roles of various health care providers are redefined in modern medicine, the integration of AI will require interdisciplinary collaboration of stakeholders in health care, which includes not only physicians but also allied health care professionals. Collecting data on a diverse mix of allied health care students is critical, as allied health care professionals make up most of the health care professionals aged <30 years in Canada [19].

### Objectives

There is currently no literature exploring the perspectives of entry-to-practice health care students on AI. This work presents the results of a nationwide survey of these students in Canada. Therefore, the goals and impacts of this survey are 3-fold. First, this work aims to explore and identify gaps in knowledge that Canadian health care students have regarding AI. This will allow us to explore the potential challenges related to knowledge acquisition of AI in health care education, and this information can, in turn, be used to inform decision-makers to better address these challenges. Second, this work aims to explore the potential differences in knowledge and perspectives on AI between students in different health care disciplines. Knowledge gaps in AI between future end users must be identified to facilitate effective communication and, in turn, improve patient safety and quality of care. Finally, this work provides an opportunity to present students’ suggestions on how to incorporate AI literacy into the health care curriculum.

## Methods

### Ethics

This prospective anonymous web-based survey study received ethics approval from the local institutional behavioral research ethics board (H20-03339). Participants were informed at the beginning of the survey that the survey completion would imply their informed consent.

### Study Cohort

The inclusion criteria were being aged ≥18 years and being currently enrolled in a Canadian entry-to-practice health care program at the time of this study [20]. We excluded responses from students studying outside Canada or those not in an entry-to-practice program.

### Survey Design

The survey was developed from a narrative literature review of topics in attitudinal surveys on AI [15,16,18,21,22]. Attitudinal questions such as Likert scale belief questions were adopted from previous surveys directed toward radiology residents and US citizens [16,21]. The survey was piloted within a small group from the same university, involving 5 MD students, 2 occupational therapy (OT) students, and 2 clinicians (neurologists and occupational therapists). Questions were revised for clarity according to feedback from the pilot group. The final survey comprised 15 items, including multiple-choice questions, pick-group-rank questions, 11-point Likert scale items, slider scale questions, and narrative questions (Multimedia Appendix 1) and was available in both English and French. Respondents were first asked to provide their own definition of AI and then given the following definition of AI to refer to for the remainder of their responses: “software that can learn from experience, adjust to new inputs, and make decisions” [23]. The survey focused on six broad topics: (1) demographics information, including the institution of training, program, age, gender, and level of education; (2) self-reported perceived understanding of AI; (3) attitudes toward the impact of AI on the respondent’s field; (4) whether the respondent wanted basic literacy in AI to be incorporated into their program’s curriculum; (5) priorities in AI literacy education; and (6) the settings and amount of time the students were willing to spend to acquire basic AI literacy.

### Survey Distribution

We used snowball and convenience sampling methods [24] by distributing an email with a description and a link to the web-based survey to representatives from 18 Canadian schools (Table 1). Allied health programs were selected from the Health Care Provider Taxonomy [20]. Respondents also had the option to choose *other* for the program. Any *other* programs with >20 respondents were included for analysis (eg, midwifery). The survey was hosted on an institutional survey platform (Qualtrics). Representatives were asked to distribute the survey among their student bodies. For example, at our home institution, the survey was distributed by the Faculty of Medicine, after internal approvals, to all the currently enrolled undergraduate MD students via the school mailing lists (a pool of 1152 students). For all institutions, 1 to 2 reminders were sent to the students 1 month after initial contact. Participation in this anonymous survey was voluntary and incentivized with a random draw for a gift card. Data were collected from January 2021 to June 2021.

**Table 1:** Survey respondent demographic statistics (N=2167).

Characteristic		Values, n (%)
**Gender**		
	Female	1355 (62.53)
	Male	805 (37.15)
	Nonbinary	7 (0.32)
**Age group (years)**		
	21-25	1217 (56.16)
	26-30	492 (22.7)
	31-35	136 (6.28)
	36-40	71 (3.28)
	41-45	20 (0.92)
	46-50	4 (0.18)
	≥50	7 (0.32)
	<21	220 (10.15)
**School**		
	Dalhousie University	85 (3.92)
	Laurentian University	60 (2.77)
	McGill University	44 (2.03)
	McMaster University	31 (1.43)
	Memorial University of Newfoundland	20 (0.92)
	Northern Ontario School of Medicine	62 (2.86)
	Queen’s University	64 (2.95)
	University of British Columbia	438 (20.21)
	Université Laval	24 (1.11)
	Université de Montréal	21 (0.97)
	Université de Sherbrooke	18 (0.83)
	University of Alberta	296 (13.66)
	University of Calgary	143 (6.6)
	University of Manitoba	96 (4.43)
	University of Ottawa	19 (0.88)
	University of Toronto	458 (21.14)
	University of Saskatchewan	186 (8.58)
	Western University	97 (4.48)
	Other	5 (0.23)
**Program**		
	Audiology	15 (0.69)
	Dentistry	77 (3.55)
	Dietetics	1 (0.05)
	Genetics counseling	35 (1.62)
	Medical doctorate	683 (31.52)
	Medical Laboratory Science	10 (0.46)
	Midwifery	22 (1.02)
	Nursing	514 (23.72)
	Occupational therapy	249 (11.49)
	Pharmacy	159 (7.34)
	Physical therapy	217 (10.01)
	Social work	43 (1.98)
	Speech language pathology	142 (6.55)
**Year level**		
	First year	479 (22.1)
	Second year	680 (31.38)
	Third year	550 (25.38)
	Fourth year	335 (15.46)
	Other	30 (1.38)
**Highest degree of education completed**		
	Bachelor’s degree	1160 (53.53)
	Diploma or certificate	27 (1.25)
	High school	371 (17.12)
	Master’s degree	438 (20.21)
	PhD degree	166 (7.66)
	Other	5 (0.23)

### Statistical Analysis

Participant responses were included in the analysis if they completed 65% of the questions, as this completion rate indicated completion beyond demographics for the response to be meaningful. In addition, survey responses lacking programmatic information, or those which indicated non–health care fields, were excluded. Responses were checked for duplication by checking for IP addresses and response similarities. Duplicate responses were subsequently removed. Programs with <20 responses were removed from the between-program analysis. Age was categorized into the following eight groups: <21 years, 21 to 25 years, 26 to 30 years, 31 to 35 years, 36 to 40 years, 41 to 45 years, 46 to 50 years, and >50 years. For quantitative measures, the number of respondents and the percentage of total respondents were reported. The normality of AI perception distributions could not be established using the Shapiro–Wilk test (*W*=0.953; *P*<.001). Therefore, Kruskal–Wallis analyses were performed to test for differences in attitude by age, gender, year of training, previous degree, professional interests, and regional variations, with the significance level determined by *P*<.001. When significant differences were found, post hoc Conover tests with Holm-adjusted *P* values were performed to determine which groups differed from each other. All analyses were performed using Python (version 3.8, Python Software Foundation). Data management and statistical testing were conducted using the following packages: tableone [25], scikit-learn [26], numpy [27], pandas [28], matplotlib [29], scipy [30]. Our code is available on GitHub [31]. For the definition of AI, 2 members of the research team (DL and AG) with training in engineering and health sciences, respectively, reviewed all responses and classified the definitions as accurate, partially accurate, inaccurate, or do not know. The correctness of the AI definition was assessed based on the following definition of AI: “software that can learn from experience, adjust to new inputs, and make decisions” [23]. For example, a response would be marked as incorrect if it included generic responses such as “AI is anything related to computers,” partially correct if the respondent described an aspect of AI such as machine learning, and correct if the respondent described machine intelligence in any manner. Discrepancies were flagged and reconciled with a third member (MT) of the team. Thematic analysis of free-text data on sentiments toward AI was conducted manually by 2 members (DL and AG) of the research team. Themes were grouped by these 2 members, and discrepancies were reconciled with a third member of the team (MT).

## Results

### Overview

The study was initiated in December 2020, approved by the ethics board on January 25, 2021, and data were collected between January 25 and May 31, 2021. The total number of survey respondents was 2167 at the time of submission of the manuscript, and all analyses were completed using data from 2167 data points. We expect that the results will be published in spring 2022.

### Respondent Demographics

A total of 2947 responses were collected from 18 universities across Canada. Out of these 2947 responses, 780 (26.47%; 261, 33.5% because of duplicate responses, 442, 56.7% because of incompletion, 1, 0.1% because of invalid age response, and 76, 9.7% for not indicating programmatic information or not being in an entry-to-practice program) were removed from the analysis. Descriptive statistics and nonparametric statistics were generated using 73.53% (2167/2947) valid responses, representing all 10 provinces across Canada (see Table 1 for demographic details). Response rates per discipline were estimated (Table 2). There were no significant demographic differences for those with complete surveys versus incomplete surveys (ie, not providing further information other than demographics).

**Table 2:** Estimated response rate per program.

Program	Estimated total number of students in Canada across all training years^a^, N	Survey sample and estimated survey representation, n (%)
Dentistry	1695	77 (4.5)
Genetics counseling	40	35 (87.5)
MD^b^	10,179	683 (6.7)
Midwifery	338	22 (6.5)
Nursing	9746	514 (5.3)
Occupational therapy	2058	249 (12.1)
Pharmacy	4610	159 (3.4)
Physical therapy	2106	217 (10.3)
Social work	4052	43 (1.1)
Speech language pathology	749	142 (19)

^a^The total number of students was estimated from enrollment statistics from the 18 schools included in the study.

^b^MD: Doctor of Medicine.

### General Attitudes and Knowledge

When asked to define AI, more than half of the respondents did not know what AI was (1107/2167, 51.08%) or had an inaccurate understanding of it (676/2167, 31.2%; Multimedia Appendix 2). Results stratifying respondents with and without an accurate understanding of the definition of AI are described in the *Post Hoc Analyses* section. Following the first open-ended question asking participants to define AI, the rest of the responses were based on the following definition of AI provided to the participants: “software that can learn from experience, adjust to new inputs, and make decisions” [19]. Overall, most reported a positive outlook on the development of AI in their respective health care fields, believed that AI would have an impact on their careers, and predicted integration of AI in their fields within the next 5 or 10 years (Multimedia Appendices 2-5).

**Figure 1:**
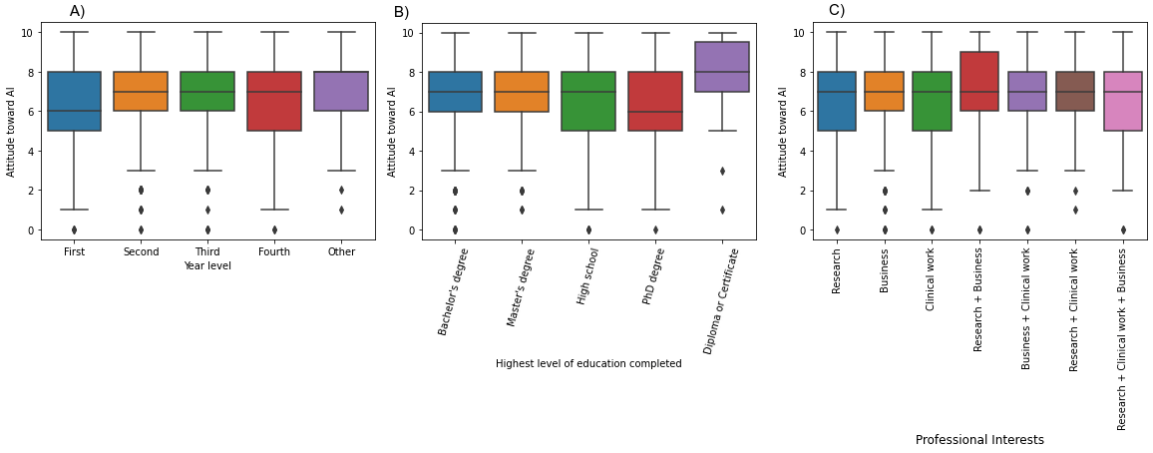
Attitude toward artificial intelligence (AI) stratified by (A) current year of study, (B) highest level of education completed, and (C) professional interests.

Using the Kruskal–Wallis test, no statistically significant differences were found in attitude toward AI between the different age groups (*H*=12.35; *P*=.09), gender (*H*=4.76; *P*=.09), or region (*H*=9.007; *P*=.61) groups. Statistically significant differences were found in attitudes toward AI for participants from different groups based on their year of training (*H*=21.359; *P*<.001), degree of education completed (*H*=32.35; *P*<.001), program (*H*=103.82; *P*<.001), institution of training (*H*=44.06; *P*<.001), and professional interests (*H*=41.08; *P*<.001). Students who were less advanced in their training had less favorable outlooks toward AI than upper-year students (*P*<.001; Figure 1). Students who had already completed a bachelor’s or master’s degree had more positive outlooks on AI than students who had completed high school only (*P*<.001) or had a PhD degree (*P*=.004). Respondents interested in pursuing research or business as part of their careers had a more favorable attitude toward AI than those wishing to focus on clinical work (*P*<.001).

**Figure 2:**
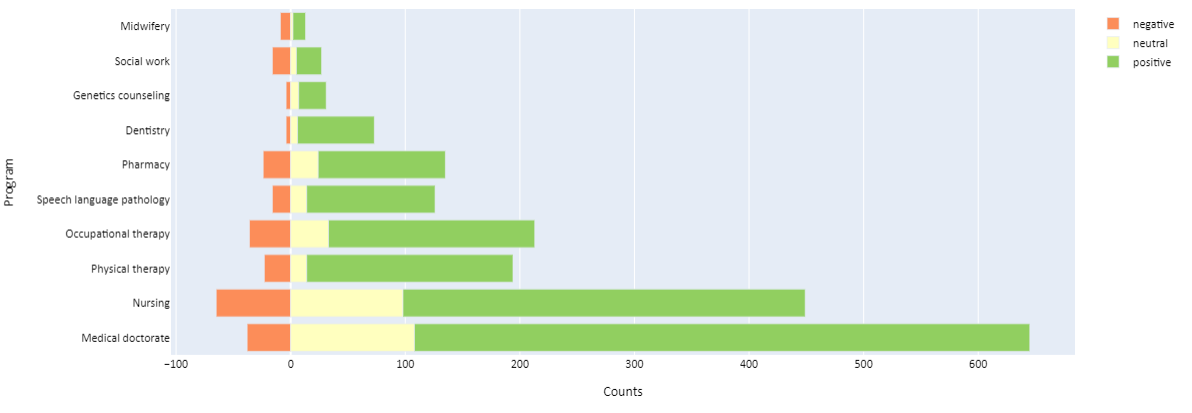
Attitude toward artificial intelligence (AI) by program or profession.

Students in medicine, dentistry, and physical therapy (PT) had similar positive outlooks regarding AI development in their fields, differing statistically from those in most other health care fields, such as genetics counseling, midwifery, nursing, OT, pharmacy, social work, and speech language pathology (SLP; Figure 2).

**Figure 3:**
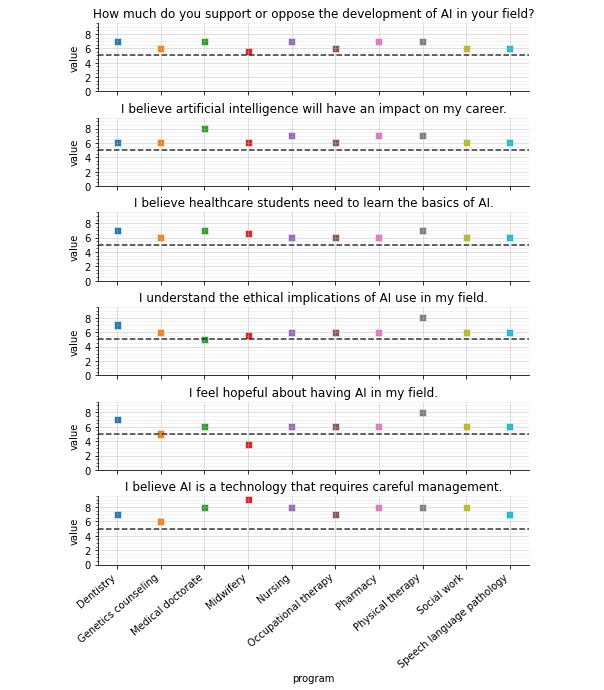
Responses to Likert scale questions by program. AI: artificial intelligence.

Overall, students in different health care programs differed in their opinions on whether AI would affect their careers (*H*=136.82; *P*<.001; Figure 3). Students studying medicine were much more likely to agree that AI would have an impact on their careers than other health care students (*P*<.001). Regardless of the health care program, students believed they needed to gain basic literacy in AI (Multimedia Appendix 3). On the basis of Likert scale self-rated responses, students differed in their self-rated understanding of AI ethics (*H*=127.705; *P*<.001). Students in PT and dentistry programs ranked higher in their perception of understanding of the ethical implications of AI than other health care students (*P*<.001), whereas students in medicine and midwifery ranked lower in their understanding of the ethical implications of AI in their fields (*P*<.001).

In terms of hopefulness toward AI development, students differed in their opinions by health discipline (*H*=98.382; *P*<.001). Specifically, students in midwifery felt significantly less hopeful than other health care students (*P*<.001), whereas students in dentistry and PT were more hopeful about AI than other health care students (*P*=.009). Students in PT and dentistry were most worried about the development of AI in their fields (*P*=.002), whereas students in midwifery were least worried about the development of AI in their field compared with other health care students (*P*=.003). Most students (1942/2167, 89.62%) from all health care fields believed that AI was a technology requiring careful management, with students in medicine, nursing, pharmacy, and midwifery sharing stronger views on this than other health care students (*P*<.001).

### Thematic Analysis

Thematic analysis of responses to the question “use one word or sentence to describe how you feel about AI in your field,” revealed three key themes: cautious optimism, uneducated and uncertain, and concerns about being replaced by AI.

#### Cautious Optimism

Across all 10 health care programs, there were respondents who expressed optimism and hopefulness toward AI. Students believed that “[AI] will greatly improve the practice of medicine to be more efficient and reliable” [an MD student] and “[it] could prevent mistakes and increase efficiency” [a midwifery student]. There was a sense that “It may be inevitable for AI to be involved in my field to some degree in the future” [a social work student]. Caution was expressed in conjunction with these responses as students were aware that “AI needs to be developed and implemented carefully as to not take away the individualization of...healthcare” [a midwifery student].

MD students generally had more positive sentiments toward AI. Many of them would support the development of AI if patient outcomes could be improved:

If it helps patient outcomes, I’m in.

an MD student

Another MD student mentioned the following:

If AI is used for enhancement of the field rather than replacement of skilled workers then my comfort increases, however I am apprehensive of the potential misuse of the technology and the risk of job loss to physicians.

Nursing students were generally optimistic toward AI and had more comments on risks associated with AI than those from other programs:

AI can pose huge confidentiality issues for patient healthcare records.

a nursing student

Another nursing student stated the following:

I think it can have great impacts but still need to be monitored for safety.

Students in OT and PT frequently mentioned AI as a tool that will “do boring repetitive things for humans” [an OT student]. One of the PT students mentioned that AI will “replace high-risk treatments.” Students in both programs agreed that AI would “greatly improve work efficiency” [an OT student].

Dentistry students generally had more positive attitudes toward AI. Most responses were single words, such as “good” and “exciting.” Some students commented that “[AI is] the trend of future development.” Genetics counseling students did not have program-specific variations from the thematic analysis.

Although there were students in all health care programs who opposed the development of AI in their fields, some social work students more strongly voiced this in their responses that there is “no role [for AI].” Another student said, “It’s a threat to my profession.” However, the main ethos from social work students aligned with the general theme of cautious optimism.

Students in midwifery had more negative attitudes toward AI:

I believe that AI has already had a negative impact in my field.

Another student mentioned the following:

It does not have a place in midwifery, you cannot teach empathy and comfort measures for a woman in labour.

This was echoed by another student who said, “It would probably make parts of my job more complicated.” More frequently occurring words used by midwifery students to describe feelings toward AI included “scared,” “dangerous if not careful,” and “apprehensive.”

#### Uneducated and Uncertain

Permutations of this quote frequently appeared in the responses:

I feel under-informed and under-educated.

an OT student

Many students expressed feeling “unsure,” “uncertain,” or “there is no feeling” toward AI (PT students). Health care students also expressed being uncertain about how AI would be applicable to their fields, if at all:

I don’t know if AI would be applicable in Speech pathology.

an SLP student

I don’t see [AI] having a huge impact in pharmacy in the near future.

a pharmacy student

I believe it will be able to significantly help with dental lab work but don’t see much clinical applicability.

a dentistry student

I don’t really care because I don’t understand how it applies to me.

an MD student

Students also reported not having given much thought to the idea of AI in their fields. A nursing student stated the following:

I have not learnt much of AI and how it can be used in nursing, this survey has sparked my interest and it is something I am going to read up on.

A pharmacy student also mentioned the lack of opportunities to learn more about AI in their fields:

I always hear about it but I don’t see a lot of opportunities to learn about it.

Regarding having to learn about AI, MD students reported concerns over having to learn more on top of their already intensive curriculum:

It scares me that MD students/healthcare professionals on top of everything else will one day have to learn AI, this is similar to learning statistics to be able to do research properly. It is simply not our field and expertise and it stresses me out.

SLP students were doubtful about the role that AI will play in their field. One of the students mentioned that they were “doubtful about the role AI could even play.” Others went into details about the inapplicability of AI:

I don’t think AI is going to be beneficial/used in the field of speech and language considering the current limitations relating to automatic speech recognition (AI can’t easily recognize “atypical” speech).

Another student stated the following:

I think AI may play a large role in training future clinicians, but not in clinical work or practice.

#### Concerns About Replacement

Even when unprompted, respondents often cited aspects of their jobs that cannot be replaced by AI:

Although helpful, it can’t replace human emotion.

a nursing student

A genetics counseling student stated the following:

I feel that as I am in a counselling field that emphasizes human emotion, AI is not very relevant. It may be useful for the technological pipelines that generate and interpret genetic information but won't be able to fill the role of a genetic counsellor.

Worries about job loss were a common theme among health care students:

I am a bit worried I may get replaced.

a social work student

I hope AI doesn’t completely devour my field.

a pharmacy student

I am nervous it will replace jobs and that it may have negative ethical implications.

a nursing student

Pharmacy students shared many examples of how AI could be applied in their fields:

I think AI has the potential to take some of the technical work off of pharmacists and allow us to focus on more clinical work—exciting potential.

It can be helpful when dispensing high-volume medications in a timely manner, especially when short-staffed.

Many also shared concerns over job replacement:

I think AI is emerging in the pharmacy world and can simplify a lot of routine jobs but also has the potential to take over a lot of human tasks which could be concerning for the job market in the future.

Another student emphasized that although AI has applications in pharmacy, it should not replace jobs:

[AI] may be useful to carry out technical tasks but all clinical work should be carried out by professionals. AI should [be] an aid, not a replacement of labour.

### Curriculum Integration

Over half of the respondents (1373/2167, 63.36%) believed that gaining basic literacy in AI should be part of their curriculum. Importantly, this sentiment was shared with the cohort of students (235/2167, 10.84%) who opposed the development of AI in their fields. In this cohort, 44.7% (105/235) believed that health care students needed to learn the basics of AI and that it should be within their program curricula. Regarding how AI literacy should be incorporated into their programs, respondents preferred either a multiple-workshop series (638/2167, 29.44%), 1- or 2-hour workshops (501/2167, 23.12%), and a 1-day course (349/2167, 16.11%). A minority (148/2167, 6.83%) expressed interest in pursuing graduate-level education to learn more about AI. The rest of the respondents felt that a combination of the above would be sufficient (531/2167, 24.5%; Multimedia Appendix 4).

**Figure 4:**
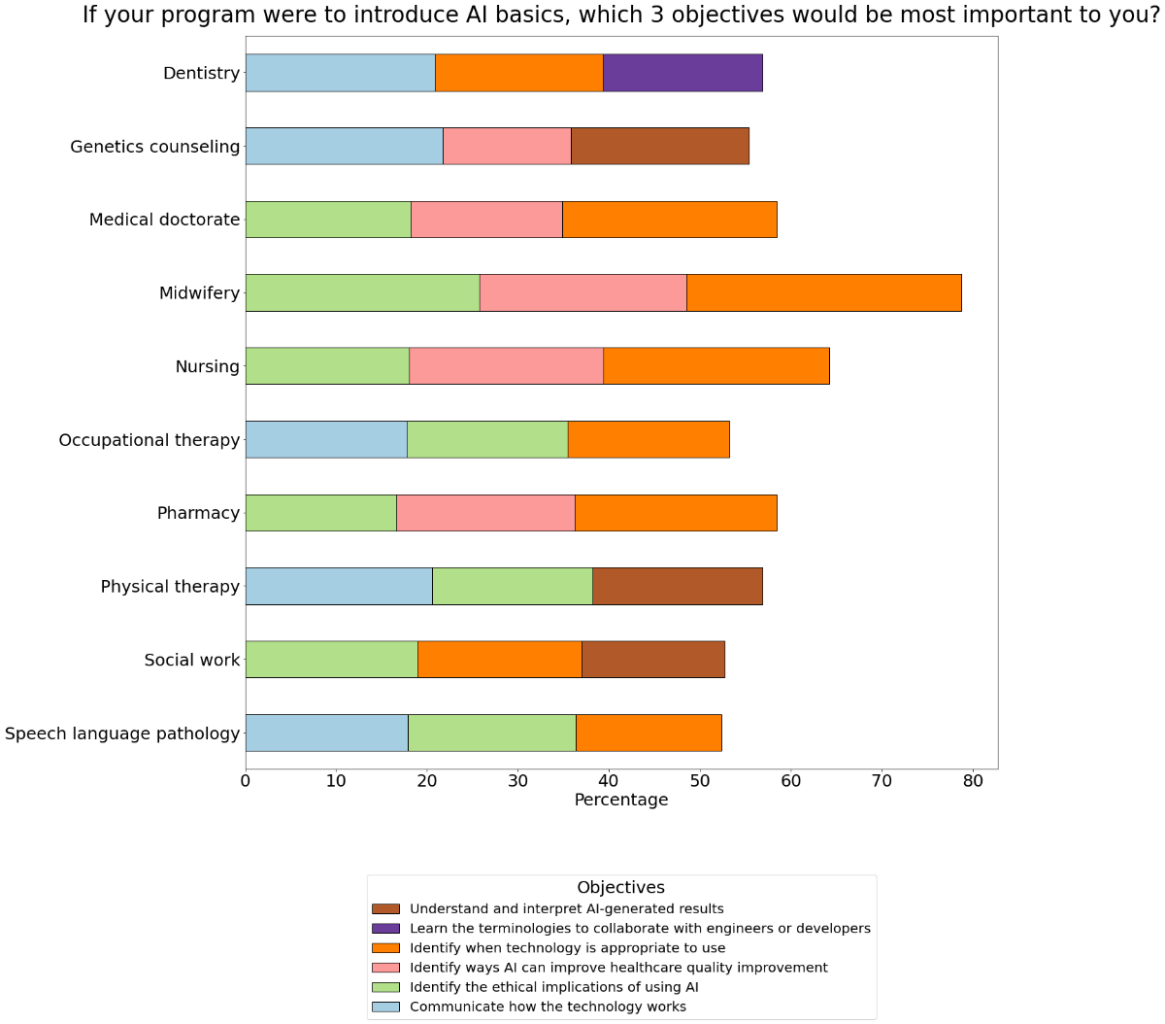
Top 3 objectives ranked important per program.

When asked to rank important objectives that should be covered in AI literacy education, the following three objectives (in order of ranked importance) were most frequently selected by respondents: *identify when technology is appropriate for a given clinical context*, *identify the ethical implications of using AI in the clinical context*, and *identify ways AI can improve health care quality improvement* (Figure 4). For those who chose *other* objectives, some wanted to learn how AI may affect billing and patient turnover as well as data privacy, security, and legal issues related to AI use. See Multimedia Appendix 5 for all objectives ranked per program.

### Post Hoc Analyses

Only a minority of respondents (338/2167, 15.6%) provided a correct definition of AI. To better understand how survey responses differed by familiarity with AI, we assigned respondents within our sample, post hoc, to one of three levels based on their initial definitions of AI: *low* (unable to provide a definition of AI), *intermediate* (provided an incorrect or partially incorrect definition of AI), *high* (provided a correct definition of AI). We repeated our quantitative analyses while stratifying by AI familiarity.

**Figure 5:**
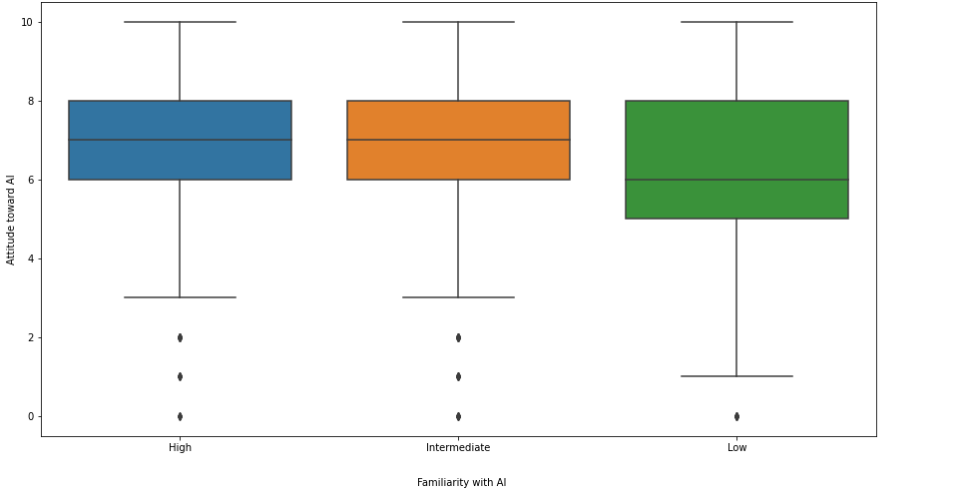
Attitudes toward artificial intelligence (AI), stratified by familiarity with AI.

Students with high and intermediate familiarity had a more positive outlook (*P*<.001; Figure 5) than those with low familiarity (*P*<.001). Students with high familiarity also believed that AI would have an impact on their careers sooner than their peers with less familiarity with AI (*P*=.002). Students with a low level of familiarity were more likely to indicate that AI literacy should be part of their curriculum when compared with students with a high level of familiarity (*P*<.001).

Qualitative findings stratified by AI knowledge similarly mirrored findings from the quantitative analysis: students who responded with “I don’t know” when asked to define AI were more likely to feel uncertain and cautious toward AI. Common sentiments in this subgroup included “I’m not sure what to expect,” “Concerning if it would completely replace nurses,” and “Afraid it will make some future careers in medicine obsolete.” Subgroups that ventured into a definition for AI (regardless of whether the responses were correct, incorrect, or partially correct) did not differ markedly from the general sentiment analysis.

## Discussion

### Principal Findings

This is the first study to investigate the views of health care students from different health care programs on AI in health care. We found that health care students generally held cautious optimism toward AI in their fields, although more than half of the health care students indicated not knowing what AI was or how it may be relevant in their fields. Overall, we found that health care students felt unprepared and uneducated about AI, which may have contributed to their fear and anxiety over this topic. This study is distinct from previous work surveying MD student perspectives on AI in health care [16,18,32] because of its size, nationwide cohort, and scope across different health care disciplines.

Consistent with findings from other MD student or resident surveys on AI [15,18,33], we found that health care students had limited knowledge of AI. The lack of understanding of AI indicates an urgent need for education, as health care providers may increasingly need to use AI applications in their practices. Not understanding how AI may be integrated into their fields or how to interpret AI-generated results may hinder care delivery and lead to fear or distrust of such applications [15,16,34]. A total of 2 previous surveys assessing medical students’ self-perceived understanding of AI also found limited AI-related knowledge among respondents. A 2020 European survey showed that only one-third of the medical students surveyed stated that they had a basic knowledge of AI [16]. A 2021 survey of medical students in Ontario showed that respondents believed that they understood what *AI* meant; however, when asked about specific terminologies related to AI, such as machine learning or neural networks, students did not understand them [22]. A major limitation of these studies was that knowledge of AI was assessed using the self-reported perception of AI understanding using Likert scales. Our study offers the first glimpse into a less subjective view of health care students’ understanding of AI. Over half of the survey respondents indicated that they did not know what AI was. This finding suggests that our sample differed widely in their knowledge of AI. In addition, the respondents’ reported perceptions of AI might vary systematically with their knowledge of AI.

We identified that most Canadian health care students felt equally hopeful and worried about the role of AI in their fields, which may be related to a lack of understanding of AI. A similar mixed sentiment was previously expressed by MD students from the United Kingdom [16] and by practicing health professionals in France and the United States [33,35]. Health care workers appear hopeful that the incorporation of AI will bring improvements in diagnostic accuracy [34] and patient monitoring [33] and reductions in medical errors [36] and improve the accessibility of care in medically deficient regions [33]. Worries regarding the incorporation of AI into health care may be attributable to the potential for replacement of health professionals [15] and additional knowledge requirements in their fields [37]. These ideas were reflected in our respondents’ willingness to adopt any intervention that could support patient care while also expressing concerns about future employment and lack of knowledge regarding AI.

Attitudes toward AI differed among the following demographic variables: (1) year of training, (2) highest degree of education completed, (3) university, (4) professional interests, and (5) profession. Students in the earlier years of their training had less favorable outlooks toward AI than upper-year students. The increased clinical exposure in later years of training [38,39] may explain this finding, where having observed how technology is used in situ may have dispelled misconceptions about the clinical utility of technology [38,39]. This survey also identified that students who had completed a bachelor’s or master’s degree had a more positive outlook on AI than students who had completed high school and, interestingly, a PhD. In fact, students with high school as their highest level of education did not differ significantly from students who had completed a PhD. There is evidence to suggest that there is a larger emphasis placed on AI application in higher education, with most research focusing on undergraduate students over postgraduate students [22]. In terms of professional interests, students who wished to pursue research or entrepreneurship in their careers had a more favorable outlook on AI than those who wished to focus on clinical work. Laï et al [33] previously found that industry professionals and researchers are the driving force for AI implementation as these individuals are mainly focused on development, whereas clinicians consider themselves as users of AI and tend to remain more pragmatic, especially when many have yet to see AI applications being used successfully and ethically in clinical medicine. Health care students who are interested in research and business may have experience in these fields and, therefore, may have a line of thinking that is more closely aligned with developers. This contrasts with students mainly interested in clinical work, who may have more uncertainties regarding AI in health care or fear that AI may be applied at the expense of human connections in disciplines with an emphasis on direct patient interactions.

When asked which 3 objectives would be most important to include when introducing AI basics to their educational programs, students from different disciplines ranked the objectives differently. Although the exact order of the objectives might have differed slightly among the different professions, most professions consistently ranked the following among the top objectives: *identify what technology is appropriate for a given clinical context*, *identify the ethical implications of using AI in clinical contexts*, and *identify ways AI can improve health care quality improvement*. This may indicate that basic AI programs should focus on these 3 objectives to meet the needs of student interests from multiple disciplines. Of note, dentistry students differed from other health care students in that 2 of the top-ranked objectives involved *communicating how the technology works* and *learning the terminologies to collaborate with engineers*. This may be because dentistry is a largely private practice in Canada, and thus, dentistry students are more business- and relationship-oriented. Rehabilitation professionals (OT, PT, and SLP) ranked *communicating how the technology works* as one of the top-ranked objectives, which may reflect their priority to communicate the underlying technology in ways that strengthen therapeutic alliance, as therapeutic alliance and rehabilitation adherence were found to be positively correlated [40,41]. Genetics counseling students were especially interested in learning about interpreting AI-generated results, which was not surprising, given that this profession involves interpreting genetic testing results.

The results revealed that across all health care programs, respondents felt uneducated about AI in general. This may have to do with the fact that although some Canadian health professional institutions include AI objectives in their core curriculum, many others do not [32,42]. When asked to comment on their feelings toward AI, students in medicine and nursing were generally optimistic toward the role of AI, whereas students in midwifery, for example, had more negative attitudes. A possible reason for these differences can be explained in the study by Doğaner et al [43], which found that health science students feel that although AI will benefit technology and health, it will negatively affect employment and sociology. These findings were reflected in the student responses in our study, as most negative responses revolved around the themes of employment and the fear of losing human connections. These feelings may be because of a lack of understanding of AI but should not be discounted; further research should be conducted to address these apprehensions [16].

With most health care students predicting that AI would be integrated within their field in the next 5 to 10 years, the lack of introduction to AI in health care curricula is especially striking. Fears and misconceptions related to AI replacing health care professionals could be addressed and prevented by introducing AI into health care education. Regardless of the differences in attitudes among students from different professions, students will benefit from additional education on the topic. Although to our knowledge, no work to date has focused specifically on the best ways to deliver AI literacy in health care education, students may share similar learning preferences to statistics literacy. For instance, previous work suggests that health care students prefer that statistics be incorporated into their education by using a variety of media [44] using content that will be relevant to their future practice [44,45]. Previous investigators have explored a variety of delivery formats, including blended [46], problem-based [47], competition-oriented [48], and a mix of lecture and seminar [44] models in statistical education. Future research should investigate how insights into statistical education gleaned from research can be applied to AI literacy education.

An AI-friendly health care curriculum is essential as future health care providers will likely be responsible for the oversight of algorithmic interpretation of patients’ health care data [49]. Beyond the health care curriculum, the integration of AI into clinical practice must be carefully evaluated. Gaps in knowledge among end users of AI applications in different health care disciplines must be identified to ensure the safety and effectiveness of AI applications in patient care. This is necessary, given the interdisciplinary nature of AI, as well as the current disconnect in the level of understanding between health care professionals and their computer science colleagues. Future end users must be digitally competent and confident in data literacy and their ability to use and interpret AI applications. Therefore, it is imperative to include a basic understanding of AI in health care education, as health care students represent the future generation of AI end users.

The limitations of this study include recruitment and participation bias. Recruitment was conducted by MD student representatives from Canadian medical schools, potentially limiting recruitment from other health care programs. There was less representation from institutions that did not have MD programs, except Laurentian University. There was also less representation from men, those aged >40 years, and those from rural and territorial regions. This is important as AI in health care can potentially improve equity and access; however, equitable representation needs to be in place for its success. Those who already have interest, knowledge, or participation in AI may have been more inclined to participate in the study. Furthermore, responses on perceived understanding, attitudes, and perceptions of AI may be biased by the degree of exposure to AI in the respective fields and institutions. Compared with the number of physicians and allied health professionals in the workforce [19], MD students were overrepresented in the study, whereas nurses and allied health workers were underrepresented.

### Conclusions

This study adds to the current literature on health care students’ attitudes toward AI and their learning preferences and self-identified areas of knowledge gap. Canadian health care students were cautiously optimistic about the role of AI in their fields; however, many felt uneducated about this topic. Health care students in different programs identified different curricular needs, and such program-specific needs should be considered with the curriculum integration of AI. The findings from this nationwide survey contribute to our understanding of knowledge gaps in AI among students and will advance education across different health care professions.

## Appendix

Multimedia Appendix 1 Full survey.

Multimedia Appendix 2 Respondent familiarity with artificial intelligence.

Multimedia Appendix 3 Respondent belief on artificial intelligence curriculum integration.

Multimedia Appendix 4 Respondent preference on artificial intelligence education format.

Multimedia Appendix 5 Objectives ranked important per program.

## Abbreviations

AI: artificial intelligence
DL: deep learning
MD: Doctor of Medicine
OT: occupational therapy
PT: physical therapy
SLP: speech language pathology
