# Supplementary material for: Health Care Students’ Perspectives on Artificial Intelligence: Countrywide Survey in Canada
Source: JMIR Med Educ. 2022 Jan 31;8(1):e33390. doi: 10.2196/33390 (PMC8845000; doi:10.2196/33390)
Supplement: Multimedia Appendix 1 [file mededu_v8i1e33390_app1.docx]

# Supplementary Documents

Full Survey

1. Which school are you currently attending?

o UBC

o University of Calgary

o University of Alberta

o University of Manitoba

o University of Saskatchewan

o University of Toronto

o McGill University

o Dalhousie University

o McMaster University

o Memorial University of Newfoundland

o Northern Ontario School of Medicine

o Queen's University

o Universite de Montreal

o Universite de Sherbrooke

o Universite Laval

o University of Ottawa

o Western University

o Other ________________________________________________

2. Which program are you enrolled in?

o MD

o Physical Therapy

o Occupational Therapy

o Speech Language Pathology

o Nursing

o Pharmacy

o Genetics counselling

o Dentistry

o Social work

o Other ________________________________________________

3. What year of the program are you in?

o 1st year

o 2nd year

o 3rd year

o 4th year

o Other ________________________________________________

4. What is your age?

________________________________________________________________

5. What is your gender?

o Female

o Male

o Other ________________________________________________

6. What is the highest degree of education you have completed?

o High school

o Bachelor's degree

o Master's degree

o PhD degree

o Other ________________________________________________

7. In general, which of the following apply to you? (can select multiple)

▢ I would like to do research as part of my career in the future

▢ I would like to start my own practice/business in the future

▢ I want to focus on clinical work only

8. Question 1: Describe Artificial Intelligence (AI) in one sentence. Please enter "Don't know" if applicable.

________________________________________________________________

For the purpose of this survey, we define artificial intelligence (AI) as "software that can learn from experience, adjust to new inputs, and make decisions".

9. On a scale of 0 to 10, how much do you support or oppose AI development in your field of study:

0 = strongly oppose 5 = neutral 10 = strongly support

0 1 2 3 4 5 6 7 8 9 10

10. On a scale of 0 to 10, please rate the extent that you agree with the following statements:

0 = disagree completely 5 = neutral 10 = agree completely

0 1 2 3 4 5 6 7 8 9 10

- I believe artificial intelligence will have an impact on my career.
- I believe healthcare students need to learn the basics of AI.
- I understand the ethical implications of AI usage in my field.
- I feel hopeful about having AI in my field.
- I am worried about the role AI will play in my field.
- I believe AI is a technology that requires careful management.

11. Please use one word or sentence to describe how you feel about AI in your field:

________________________________________________________________

12. If your program were to introduce AI basics, which 3 objectives would be most important to you?

Drag and drop here

______ Identify when technology is appropriate for a given clinical context

______ Understand and interpret AI-generated results

______ Be able to communicate how the technology works in a way that others can understand

______ Identify the ethical implications of using AI in clinical contexts

______ Understand how the underlying technological processes work

______ Learn the terminologies in order to communicate and collaborate with engineers/developers

______ Identify ways AI can improve healthcare quality improvement

______ Other:

13. How soon do you think AI will impact your career?

o in 5 years

o in 10 years

o in 20 years

o in 50 years

o not in my lifetime

14. Should learning about AI basics be part of your curriculum, or should it be outside of curriculum time (extracurricular)?

o Should be part of my program/curriculum

o Should be outside of curriculum time

o Other ________________________________________________

15. Which of the following would you be interested in attending to learn more about AI basics? (can select multiple)

▢ 1-day course

▢ Multiple workshop series

▢ 1- or 2-hour workshop

▢ Graduate-level education (Master's, PhD)

▢ Other ________________________________________________

16. (optional) Thank you so much for participating in our survey! If you'd like to enter the draw to win a $20 gift card to Amazon or Tim Hortons, please enter your email address below.

________________________________________________________________
