# Supplementary figures and images for: Health Care Students’ Perspectives on Artificial Intelligence: Countrywide Survey in Canada
Source: JMIR Med Educ. 2022 Jan 31;8(1):e33390. doi: 10.2196/33390 (PMC8845000; doi:10.2196/33390)

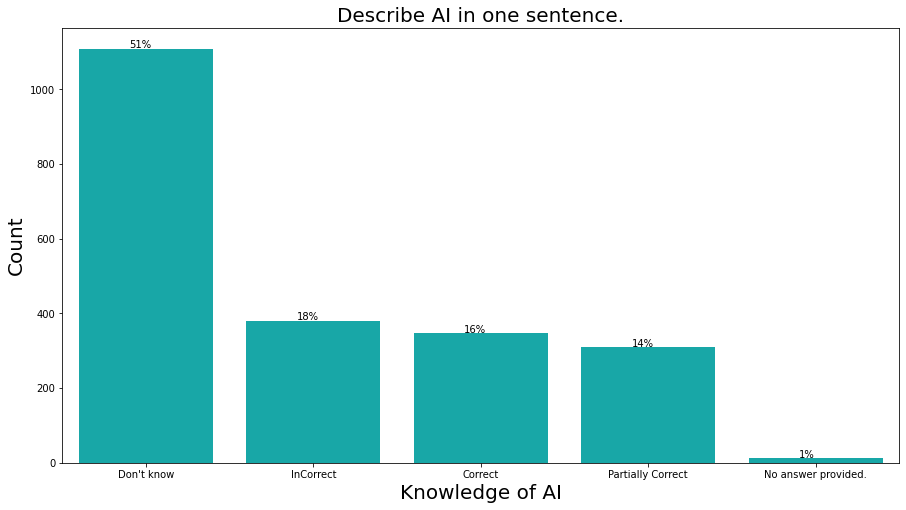

Supplement: Multimedia Appendix 2 [file mededu_v8i1e33390_app2.png]

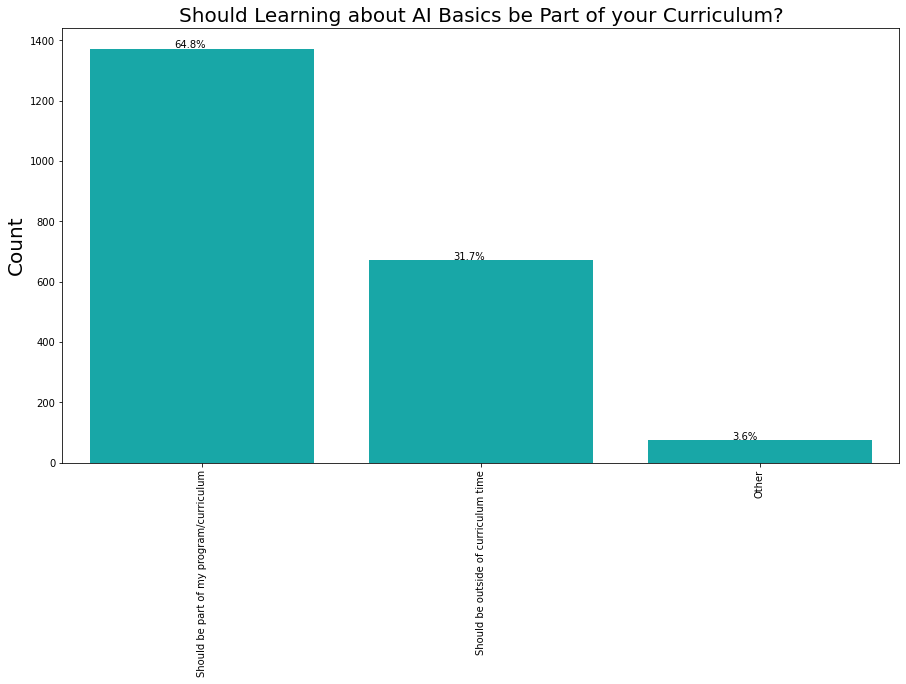

Supplement: Multimedia Appendix 3 [file mededu_v8i1e33390_app3.png]

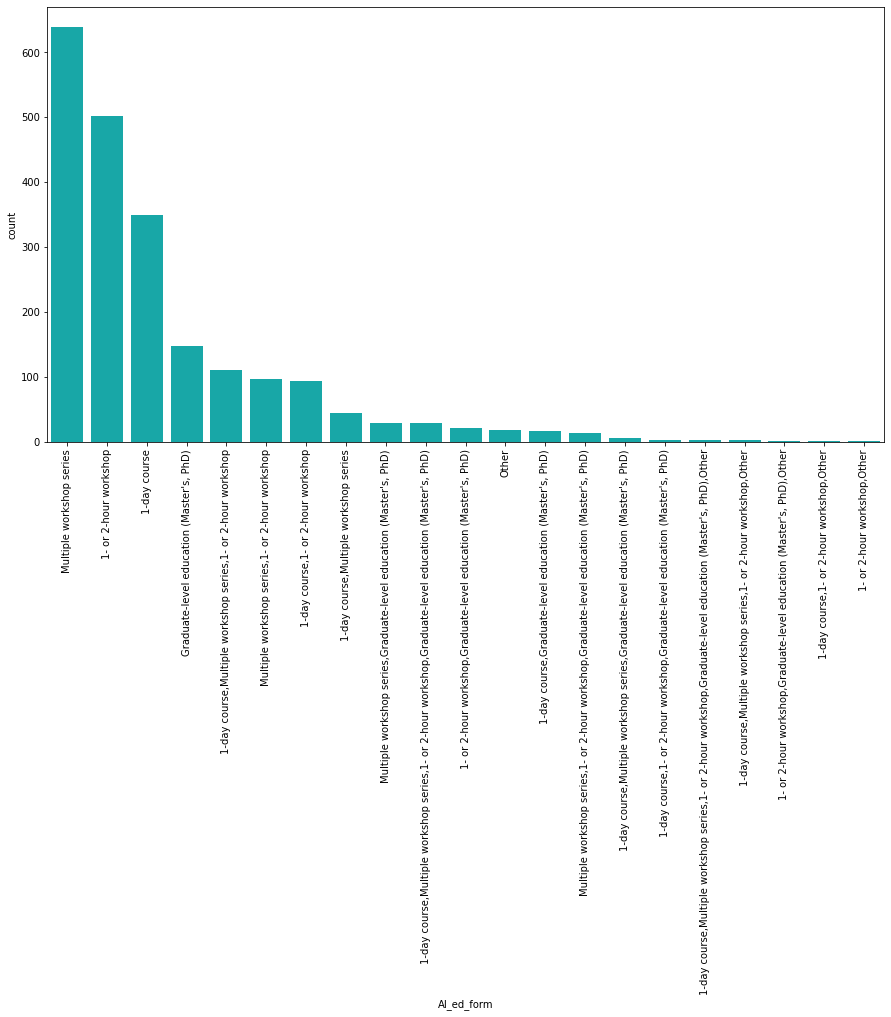

Supplement: Multimedia Appendix 4 [file mededu_v8i1e33390_app4.png]

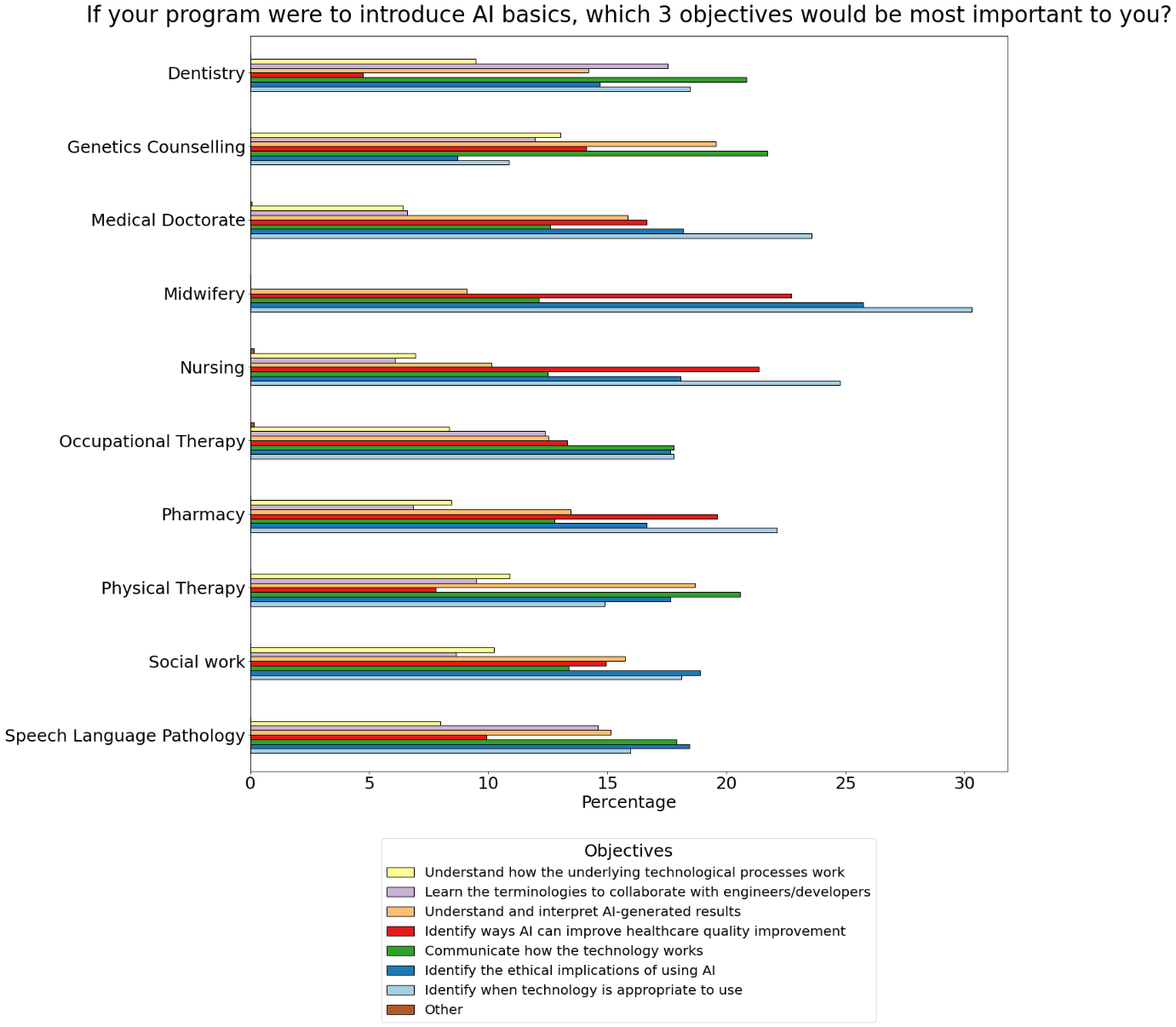

Supplement: Multimedia Appendix 5 [file mededu_v8i1e33390_app5.png]
